# Antibodies to the CD4-binding site of HIV-1 gp120 suppress gp120-specific CD4 T cell response while enhancing antibody response

**DOI:** 10.1186/1750-9378-3-11

**Published:** 2008-07-18

**Authors:** Maria Luisa Visciano, Michael Tuen, Pei-de Chen, Catarina E Hioe

**Affiliations:** 1 Department of Pathology, New York University School of Medicine and Veterans Affairs New York Harbor Healthcare System, New York, New York, USA

## Abstract

**Background:**

The binding of Abs to the CD4-binding site (CD4bs) of HIV-1 envelope gp120 has been shown to obstruct the processing and generation of helper epitopes from this antigen, resulting in poor presentation of various gp120 epitopes by MHC class II to CD4 T cells. However, the physiologic significance of these inhibitory anti-CD4bs Abs *in vivo *has remained unclear. In this study, we evaluated the immunologic effects of anti-CD4bs Abs *in vivo *using a murine model.

**Results:**

Animals were immunized with recombinant envelope proteins with or without CD4-binding activity (designated CD4bs^+ ^Env and CD4bs^– ^Env, respectively). As expected, anti-CD4bs Abs were generated only after immunization with CD4bs+ Env and not with CD4bs^– ^Env. The presence of anti-CD4bs Abs was associated with lower levels of envelope-specific lymphoproliferation in animals immunized with CD4bs+ Env. To further determine the specific role of the anti-CD4bs Abs, we immunized mice with gp120 in the presence of an inhibitory anti-CD4bs mAb or a non-inhibitory anti-gp120 mAb. The data show that the presence of anti-CD4bs mAb reduced CD4 T cell responses to gp120. However, we also detected significantly higher titers of anti-gp120 Abs following immunization with gp120 and the anti-CD4bs mAb.

**Conclusion:**

Anti-CD4bs Abs can exert discordant effects on the gp120-specific CD4 T cell and Ab responses *in vivo*, indicating the importance of these particular Abs in influencing both the cellular and the humoral immune responses against HIV-1.

## Introduction

The capacity of antibodies (Abs) to enhance or decrease antigen presentation for MHC class II-restricted CD4 T cells is well documented in the literature [1-7]. Our previous studies have demonstrated that MHC class II presentation of HIV-1 gp120 is abrogated when this antigen is bound by monoclonal Abs (mAbs) to the CD4-binding site (CD4bs) of gp120 [1,2,8]. This effect is specific for the anti-CD4bs Abs, since the other mAbs directed to other regions of gp120 cause no significant inhibition. Interestingly, inhibitory anti-CD4bs mAbs are commonly produced by chronically HIV-1-infected subjects [9,10]; these mAbs have high affinity for gp120 but poor or no neutralizing activity against primary HIV-1 isolates [8,11]. Low affinity anti-CD4bs Abs generated upon limited exposure to gp120, either during acute infection or after a short course of immunization, are not inhibitory, whereas the exceptional anti-CD4bs IgG1 b12 that mediates potent and broad virus-neutralizing activity causes only partial inhibition ([8] and Hioe et al. unpublished data).

The mechanisms by which anti-CD4bs mAbs inhibit gp120 antigen presentation to CD4 T cells have been previously investigated. Upon binding to gp120, the inhibitory anti-CD4bs mAbs do not affect gp120 uptake or transport into the acidic endolysosomes of antigen-presenting cells [8,12]. These mAbs and gp120/mAb complexes also do not directly affect the CD4 T cells. The T cells remain responsive to synthetic peptides representing already-processed gp120 epitopes, and CD4 T cells specific for other antigens, such as HIV-1 p24, Mycobacterium tuberculosis MPT-32 and 85C, and cytomegalovirus, are not affected by the mAbs or immune complexes. Instead, upon the uptake of the gp120/anti-CD4b Ab complexes by antigen-presenting cells into the endolysosomal compartments, the complexes remain quite stable at the acidic pH of the endolysosomes [8] and are resistant to proteolytic digestion by endolysosomal enzymes [8,12]. Taken together, these data support the notion that the binding of mAbs to the CD4bs obstructs gp120 proteolytic processing by antigen-presenting cells such that peptidic helper epitopes are not efficiently generated and presented on MHC class II to CD4 T cells.

It should be noted, however, that the obstructive effect of the anti-CD4bs mAbs is not simply due to steric hindrance or masking of a particular helper epitope by the mAbs. These mAbs inhibit the processing and presentation of all gp120 epitopes examined thus far, including those in the C1, C2, V2, or V3 regions [1], indicative of their global effects. Importantly, the helper epitopes are located at sites distant from or irrelevant for the binding sites of the anti-CD4bs mAbs. These findings corroborate previously reported data of Kwong et al [13] indicating that the binding of mAbs to the CD4bs, but not to other gp120 regions, induces a large entropy change in gp120, causing an overall increase in gp120 resistance to enzymatic degradation. The entropic change is also accompanied by structural and antigenic alterations as evidenced by significant increases of the mAb reactivity to the V3 loop and the N terminus of gp120 when gp120 is bound by the anti-CD4bs mAbs [14]. The thermodynamic and antigenic changes induced by anti-CD4bs mAbs can be explained by the structural data showing that, unlike most Abs which bind epitopes located in one particular domain of a protein, e.g. the V3 loop in gp120 [15-17], the anti-CD4bs mAbs, similar to CD4 and the CD4i mAbs specific for the chemokine receptor binding site, draw together both the inner and outer domains of gp120 and bind to a surface that is formed by both of these domains [13,18-21].

While the capacity of anti-CD4bs Abs to block MHC class II presentation and CD4 T cell responses to gp120 has been demonstrated *in vitro *with human CD4 T cell lines or clones [1,2,8,12], the *in vivo *effects of the anti-CD4bs Abs have yet to be investigated. The present study has been designed to examine the physiologic activities of anti-CD4bs Abs *in vivo *using a murine model. Specifically, we asked whether the presence of anti-CD4bs Abs *in vivo *affects the induction of envelope-specific cellular responses and if the alterations also influence the Ab responses to this antigen. To answer these questions, we initially compared the immunogenicity of envelope antigens that have or do not have CD4-binding activity. Subsequently, we performed *in vitro *and *in vivo *studies to test the specific role of anti-CD4bs Abs. The data from these studies demonstrate that the presence of anti-CD4bs mAbs reduces lymphoproliferative responses to gp120 *in vivo *as well as *in vitro*. However, the reduced cellular responses do not lead to diminished Ab responses to gp120; the anti-gp120 Ab levels are actually augmented in animals immunized with gp120 in the presence of an anti-CD4bs mAb.

## Results

### Immunization with envelope antigen lacking the CD4 binding site stimulates higher levels of envelope-specific T cell responses

We previously demonstrated that Ab binding to the CD4bs of gp120, which renders gp120 more resistant to proteolysis, interferes with gp120 antigen processing and prevents MHC class II presentation of the gp120 antigen to the CD4 T cells [8,12]. However, these findings are from *in vitro *experiments using human CD4 T cell lines; the *in vivo *effects of the anti-CD4bs Abs on envelope-specific immune responses have yet to be examined. To begin addressing this issue, we compared humoral and cellular responses of B10.A and BALB/c mice immunized with soluble envelope proteins with or without CD4-binding activity. One envelope protein (from Advanced Bioscience) was produced from mammalian cells chronically infected with HIV-1 IIIB; this gp140 protein binds CD4 and mAbs to the CD4-binding site on gp120 (designated CD4bs+ Env). The other envelope protein was a recombinant baculovirus-derived envelope of HIV-1 NL4.3 produced in insect cells (from MicroGeneSys); this recombinant gp140 protein has no CD4 binding activity and fails to react with mAbs to the CD4-binding site (designated CD4bs- Env) [2]. To compare the immunogenicity of these envelope proteins *in vivo*, we performed two experiments in which mice were immunized intraperitoneally (i.p.) 4x or 5x with 5 μg of each protein in either incomplete Freund's adjuvant (Experiment 1) or Ribi (Experiment 2). Envelope-specific Ab and T cell responses were determined using ELISA and ^3^H-thymidine incorporation assays, respectively. Table 1 shows the levels of Abs to the whole gp120 and to the CD4-binding site in the sera collected two weeks after the final injection. Control mice receiving adjuvant and no antigen had no Abs to gp120 or to the CD4-binding site. Serum Abs to gp120 and the CD4-binding site were consistently detected in all mice receiving CD4bs+ Env. As expected, anti-CD4bs Abs were not produced in mice immunized with CD4bs- Env, although these mice generated high titers of Abs to the whole gp120 similar to the animals immunized with the CD4bs+ Env antigen.

**Table 1:** Ab levels to whole gp120 and to CD4bs in sera of mice immunized with envelope proteins that have or lack CD4 binding activity^a^

	**Immunogen &** **Adjuvant**	**n**	**Anti-gp120 ** **(end-point titer)**	**Anti-CD4bs ** **(ID_50_)^d^**
Experiment 1^b^	No Ag & IFA	2	< 20	< 20
	CD4bs^+ ^Env & IFA	4	> 2500	310 (210–470)^e^
	CD4bs^- ^Env & IFA	3	> 2500	< 20

Experiment 2^c^	No Ag & RIBI	6	< 100	< 100
	CD4bs^+ ^Env & RIBI	5	9060 (2500–12500)	2160 (630–5900)
	CD4bs^- ^Env & RIBI	5	> 12500 (12500->12500)	< 100

^a^Sera were collected two weeks after the final immunizations and tested for reactivity with recombinant gp120_LAI _in ELISA.

^b^All mice were B10.A, except for two BALB/c in the second group. Each animal was immunized 4x i.p. with 5 μg of antigen in IFA.

^c^All mice were BALB/c and each was immunized 5x i.p. with 5 μg of antigen in RIBI.

^d^Serum dilutions that block CD4 binding to gp120_LAI _by 50%.

^e^Geometric mean and range of the Ab levels from all mice in each group.

**Figure 1:**
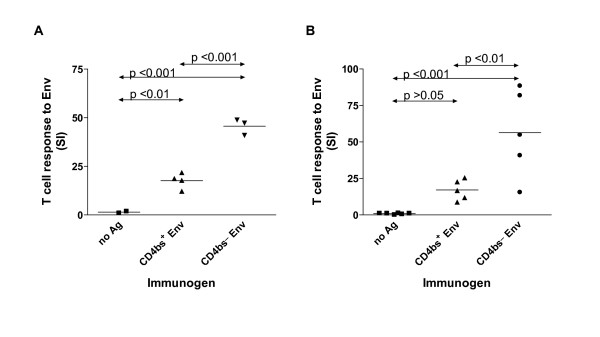
**More robust envelope specific lymphoproliferation is induced in mice immunized with CD4bs- Env as compared to CD4bs+ Env**. (A) Mice were immunized four times i.p. with CD4bs+ Env, CD4bs- Env, or no antigen in the presence of incomplete Freund's adjuvant. (B) Mice were immunized five times i.p. with the same envelope antigens and Ribi adjuvant. Splenocytes were collected two weeks after the final boost and tested for envelope-specific proliferation by ^3^H-thymidine incorporation. Proliferation in the presence of antigen (3 μg/ml) or medium alone was measured after a 5-day culture. The stimulation indices of individual mice and the mean values for each group (horizontal bars) are shown. Statistical analyses were done using one-way ANOVA with Bonferroni's multiple comparison test.

Envelope-specific lymphoproliferation was assessed by culturing splenocytes from the individual immunized mice with either medium containing envelope protein or medium alone for 5 days. Fig. 1 shows that mice immunized with CD4bs- Env had higher levels of envelope-specific lymphoproliferative response than mice immunized with CD4bs+ Env. The difference between these two groups was statistically significant and was observed with both B10.A and BALB/c mice and with the two adjuvants tested (Fig. 1A vs. 1B). Control mice receiving no antigen did not respond to the envelope protein. Lymphoproliferation was assessed in response to the same envelope antigens used for immunization (Fig. 1A) or to gp120_LAI _from a difference source (ImmunoDiagnostics) (Fig. 1B), and comparable results were obtained. These data show that immunization with CD4bs- Env induces high levels of envelope-specific Th responses, while immunization with the CD4bs+ Env antigen generates anti-CD4bs Abs and induces a poorer T cell response to the envelope antigens. However, the contribution of anti-CD4bs Abs in suppressing envelope-specific T cell responses remained unclear, since the envelope antigens used were derived from mammalian vs. insect cells and differ not only in the presence or absence of the CD4bs, but also in the extent and complexity of their glycosylation, which is known to have a significant impact on the immunogenicity of the envelope antigens [22-25].

### Monoclonal anti-CD4bs Abs suppress gp120 antigen presentation to mouse CD4 T cells

**Figure 2:**
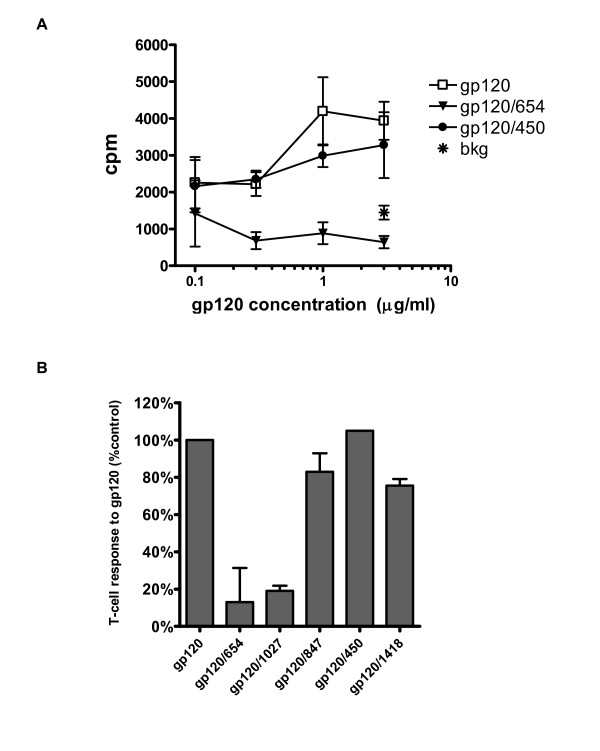
**Proliferative response of a murine CD4 T cell clone to gp120 is suppressed when gp120 is bound by anti-CD4bs mAbs**. A) Mouse T cell clone T1 was tested for proliferation to different concentrations of gp120, alone or in the presence of anti-CD4bs mAb 654 or anti-C5 mAb 450 (10 μg/ml each). B) Proliferation of clone T1 was tested in response to different gp120/mAb complexes. For comparison, the response to gp120 alone is normalized to 100%. Gp120 was tested at 3 μg/ml and combined with 10 μg/ml of each mAb. Irradiated splenocytes were used as antigen-presenting cells. Average values and standard deviation from two repeated experiments are shown.

To further examine the specific contribution of anti-CD4bs Abs in suppressing envelope-specific T cell responses, we evaluated the effects of human monoclonal anti-CD4bs Abs known to block gp120 presentation on the proliferative response of a mouse CD4 T cell clone specific for gp120. An I-E^k^-restricted CD4 T cell clone specific for an epitope in the C4 domain of gp120-IIIB was tested [26], and irradiated splenocytes from B10.A (2R) mice were used as antigen-presenting cells. The clone proliferated well in response to gp120 alone. However, similar to our findings with the human gp120-specific CD4 T cells [1,2,8,12], the response of the mouse CD4 T cells to gp120 was abrogated when gp120 was complexed with human anti-CD4bs mAb 654 (Fig. 2A). In contrast, the addition of an anti-C5 mAb (450) caused no inhibition. Subsequently, we tested other anti-gp120 mAbs for their capacity to suppress the CD4 T cell response to gp120. We prepared gp120/mAb complexes with two different anti-CD4bs mAbs (654 and 1027), an anti-C2 mAb (847) or an anti-C5 mAb (450), as well as gp120 mixed with an irrelevant mAb against the parvovirus as a control (Fig. 2B). The results showed that only anti-CD4bs mAbs 654 and 1027 inhibited the proliferation response to gp120 by more than 80%, while the other mAbs tested caused no inhibition, similar to the control mAb 1418. These results show that anti-CD4bs mAbs inhibited CD4 T cell responses to gp120 in the murine system, similar to that observed with the human CD4 T cell responses to gp120.

### Immunization with the gp120/anti-CD4bs Ab complex induces a lower gp120-specific lymphoproliferative response

**Figure 3:**
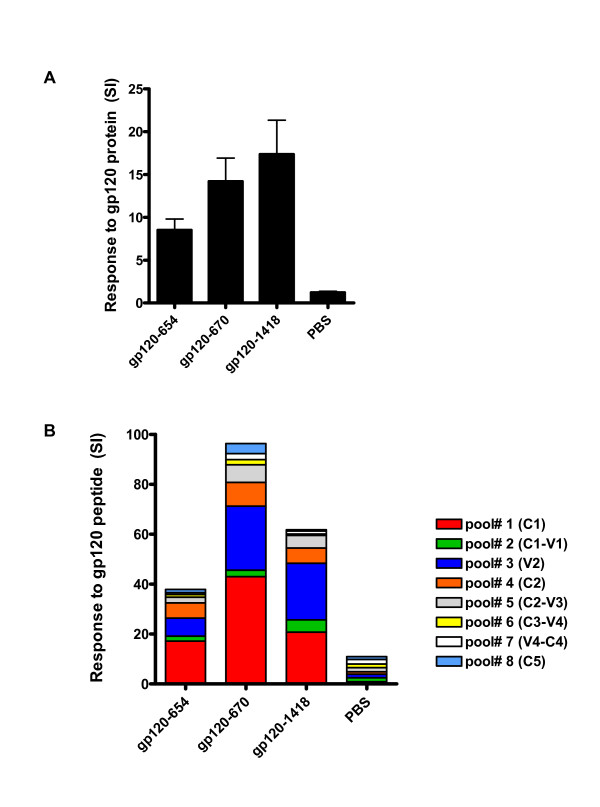
**Mice immunized with the gp120/anti-CD4bs mAb complex have lower gp120-specific lymphoproliferation than mice immunized with gp120 alone or other gp120/mAb combinations**. Mice were immunized with gp120 alone or gp120 mixed with anti-CD4bs mAb (654), anti-C5 mAb (670), or irrelevant anti-parvovirus mAb (1418), along with Ribi adjuvant. Each animal received 3 μg of gp120 and 10 μg of mAb. Control mice were injected with Ribi adjuvant containing no antigen (PBS). The splenocytes were tested in the ^3^H-thymidine incorporation assay for proliferative responses to gp120 protein (A) or pooled gp120 peptides (B).

To assess the effect of anti-CD4bs Abs on CD4 T cell responses to gp120 *in vivo*, BALB/c mice were immunized with recombinant gp120 of HIV-1 _LAI _(Progenics) in complex with an anti-CD4bs mAb (654) or with anti-C5 mAb (670). The gp120/mAb complexes were administered i.p. 4x with Ribi adjuvant. For controls, mice were immunized either with gp120_LAI _mixed with an anti-parvovirus mAb or with adjuvant only. A week after the final immunization, splenocytes were isolated from all groups and tested in the ^3^H-thymidine incorporation assay. Splenocytes from each of the groups were incubated with gp120 (1 μg/ml) or medium alone for 5 days and assessed for antigen-specific lymphoproliferation. As depicted in Fig 3A, mice immunized with the gp120/anti-CD4bs mAb complex showed a lower level of lymphoproliferation (SI = 8) as compared to mice immunized with the gp120/anti-C5 mAb complex (SI = 14) or with the control gp120/anti-parvovirus mAb mixture (SI = 17). Mice receiving only the adjuvant had no gp120-specific lymphoproliferation (SI = 1).

To determine if there are any alterations in the epitopes recognized by mice immunized with the gp120/anti-CD4bs complex, splenocytes were tested for recognition of 20-mer overlapping peptide pools encompassing the entire gp120. Each pool contained six different peptides representing a particular region of gp120, i.e. C1 (pool 1), C1-V1 (pool 2), V2 (pool 3), C2 (pool 4), C2-V3 (pool 5), C3-V4 (pool 6), V4-C4 (pool 7), and C5 (pool 8) and was tested at the final concentration of 1 μg/ml for each peptide. The results showed that in all groups of mice the highest lymphoproliferative response was directed to peptide pool 1 which represents the C1 region of gp120 (Fig. 3B). However, similar to the response to the whole gp120 protein, the anti-C1 response of mice that received the gp120/654 mAb complex was lower than those of the other two groups. The mice immunized with the gp120/654 complex also did not display significant proliferative responses to any other peptide pools tested, while the other groups showed recognition of peptide pools 3, 4, and 5, which encompass the V2 to V3 regions of gp120. Mice immunized with adjuvant only did not show any reactivity with the peptide pools (SI < 2 with each pool). These results indicate that the anti-CD4bs Abs suppress the induction of CD4 T cell responses to various epitopes on gp120, consistent with the global suppressive effects of these Abs on the processing and generation of all gp120 epitopes examined to date [1,2]. Taken together, the data indicate that the presence of Abs to the CD4-binding site of gp120 negatively affects the generation of gp120-specific lymphoproliferative responses *in vivo*, similar to the suppression seen *in vitro *on the responses of established murine and human gp120-specific CD4 T cell clones or lines.

### The presence of anti-CD4bs mAbs enhances the Ab response to gp120

**Figure 4:**
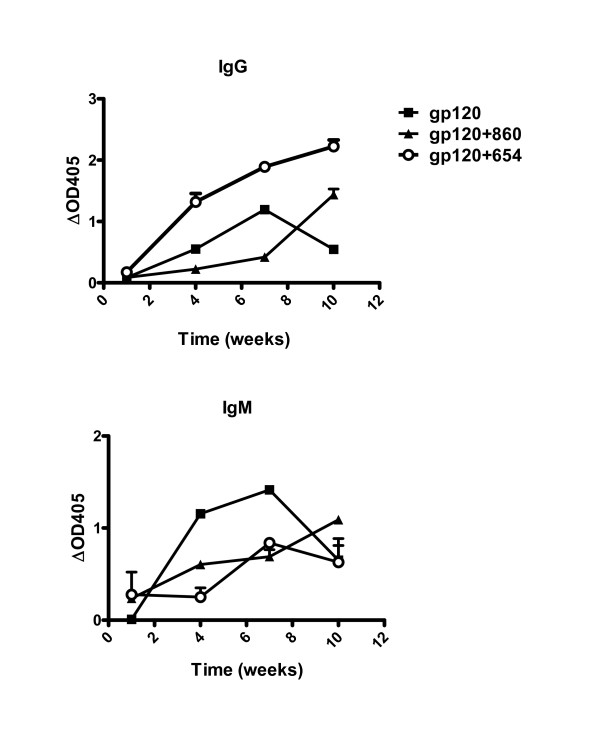
**Induction of gp120-specific IgG and IgM responses in mice immunized with gp120 or gp120/mAb complex**. Sera were collected a week after each injection and tested by ELISA at a dilution of 1:100. Sera were added to gp120_LAI _captured on the wells and gp120-specific IgG or IgM were detected with alkaline phosphatase-conjugated rabbit Abs specific for the respective mouse IgG or IgM.

Because of reduced T cell response to gp120 observed in mice immunized with the gp120/CD4bs mAb complex, we anticipated that the Ab responses to gp120 would be similarly affected. To examine this, sera collected one week after each immunization were tested by ELISA to detect the kinetics of the induction of gp120-specific IgG and IgM. Interestingly, mice immunized with the gp120/654 complex displayed faster kinetics and higher levels of gp120-specific serum IgG than mice immunized with gp120 alone or with gp120 mixed with an anti-parvovirus mAb (gp120/860) (Fig. 4). Hence, gp120/654-immunized mice had a relatively high level of gp120-specific IgG after the second immunization (OD_405 _of > 1.5), while the other groups reached a comparable level only after the third or fourth immunization. The gp120/654 complex also enhanced the induction of anti-gp120 serum IgA [14]. However, immunization with gp120/654 did not augment the induction of gp120-specific IgM production. In fact, the levels of anti-gp120 IgM in mice receiving gp120/654 and gp120/860 were comparable throughout the observation period. Administration of gp120 alone actually elicited higher levels of anti-gp120 IgM, albeit only after the second and third immunizations. These data demonstrate that immunization with gp120 in the presence of anti-CD4bs Ab alters the immunogenicity of gp120 such that the immune response to this antigen is dominated by anti-gp120 IgG, while gp120-specific lymphoproliferative responses are decreased.

## Discussion

This study provides the first *in vivo *evidence that the presence of Abs to the CD4bs of gp120 alters CD4 T cell and Ab responses to the gp120 antigen in disparate ways. These effects were specifically observed with anti-CD4bs Abs, and not with other anti-gp120 Abs examined. Hence, higher envelope-specific lymphoproliferation was induced in mice immunized with an envelope protein that lacks the CD4-binding activity and cannot elicit anti-CD4bs Abs. By contrast, lymphoproliferative responses to gp120 were lower when mice were immunized with gp120 in complex with an anti-CD4bs mAb as compared to uncomplexed gp120 or gp120 complexed with a different mAb. These data are consistent with *in vitro *data shown in Fig. 2 with a mouse CD4 T cell clone and with previously published results with a number of human CD4 T cell lines [1,2,8,12] demonstrating that the CD4 T cell proliferative responses to gp120 are suppressed in the presence of anti-CD4bs mAbs, but not other anti-gp120 or control mAbs. However, the diminished levels of gp120-specific lymphoproliferation induced in mice receiving gp120 and anti-CD4bs Abs are not accompanied by the corresponding reduction of Ab levels to gp120. As indicated by faster kinetics and higher Ab titers (Fig. 4 and [14], the Ab response to gp120 was actually enhanced in mice immunized with the gp120/anti-CD4bs complex. The enhanced Ab levels were seen in the sera with gp120-specific IgG as well as IgA, but not IgM (Fig. 4 and [14]). Moreover, we previously reported that the anti-gp120 Abs produced by immunization with the gp120/anti-CD4bs Ab complex were mainly of the IgG1 subclass [14], indicating the capacity of the anti-CD4bs Abs to skew anti-gp120 immune responses toward the Th2-type response.

Our earlier studies showed that the suppressive effects of the anti-CD4bs Abs on MHC class II presentation of gp120 antigen required a molar Ab/gp120 of at least 1 [1], indicating that formation of immune complexes is essential for suppression. Specifically, the gp120/anti-CD4bs complexes were found to be more resistant to endolysosomal proteases and other degradative enzymes, such as Endo H, than gp120 alone or gp120 complexed with mAbs to the C terminus, N terminus, or the variable loops [8,12,13]. Since CD4 T cells recognize proteolytically processed antigens, it stands to reason that the gp120/anti-CD4bs mAb complexes are poor immunogens for the CD4 T cells. Conversely, the protease-resistant gp120/anti-CD4bs complexes may serve as a more durable antigen source for B cells that recognize intact unprocessed antigens, leading to enhanced production of Abs against gp120. It should also be noted that the binding of Abs to the CD4bs of gp120 has been shown to alter gp120 antigenicity such that Ab reactivity to the specific regions of gp120, including C1 and V3, is significantly enhanced [14,27]. Consistent with these data, we observed that the enhanced Ab responses induced in mice receiving the gp120/anti-CD4bs complexes were primarily directed to the V3 loop, whereas the Ab levels to the gp120 core lacking C1, V1/V2, V3, and C5 were not increased [14].

The capacity of anti-CD4bs Abs to suppress gp120 antigen presentation to CD4 T cells while enhancing and redirecting the Ab response to the V3 loop indicates the potential role of these Abs in HIV-1 immunopathogenesis, although further studies are required to examine this issue. Studies from our lab as well as others have shown that, except for recombinant anti-CD4bs IgG b12, anti-CD4bs Abs naturally produced during HIV infection have little or no neutralizing activity against most HIV-1 primary isolates [19,28]. Hence, induction of these Abs is not likely to offer much protection against the virus. Instead, the formation of gp120/anti-CD4bs complexes during HIV-1 infection may actually contribute to further suppression of anti-viral CD4 T cell responses. Moreover, our previous studies demonstrate that Ab responses induced by these complexes were skewed toward strain-specific epitopes on the V3 loop and away from the more conserved epitopes on V3 [14,29]. Due to steric hindrance or structural alterations induced upon Ab binding to the CD4bs, the presence of these poorly neutralizing anti-CD4bs Abs also prevented the generation of more potent and cross-reactive Abs to the CD4bs or the chemokine receptor binding site [14].

Among HIV-1 infected subjects, anti-CD4bs Abs were predominantly found in the sera of progressors, while no or very low levels of these Abs were detected in long-term non-progressors or elite controllers [10]. Among the progressors, significantly higher levels of the anti-CD4bs Abs were produced by rapid progressors (with declining CD4 counts of > 53 cells/mm^3^/6 months) prior to development of AIDS than by slow progressors (with no CD4 decline in 7 years of follow-up) [9]. Notably, chronically HIV-1 infected progressors do not display significant CD4 T cell responses to envelope antigens [30,31]. Only a small percentage of HIV-1 infected subjects are able to maintain their envelope-specific lymphoproliferation; some of these subjects are long-term non-progressors and some are chronic or acute patients who received anti-retroviral therapy [10]. However, regardless of their clinical status, these exceptional individuals have undetectable or low levels of serum anti-CD4bs Abs, similar to long-term non-progressors and elite controllers. Hence, consistent with the data from the immunized mice, the presence of anti-CD4bs Abs in HIV-1-infected subjects is associated with the lack of envelope-specific CD4 T cell responses. On the other hand, CD4 T cell responses to envelope may be maintained in the absence of these Abs. It should also be noted that all of the rare HIV+ subjects with envelope-specific lymphoproliferation produce very low serum Ab titers to the whole gp120 antigen (geomean half max of 130 with a range of < 25–1500) as compared to slow or rapid progressors (geomean half max of 1500 and 1700, respectively) [10] demonstrating the discordance between CD4 T cell and Ab responses to envelope in HIV-1-infected subjects, similar to that seen in the immunized mice. By contrast, the anti-p24 Ab titers are higher in most subjects with envelope-specific lymphoproliferation than in progressors, indicating that the inverse association between CD4 T cell and Ab responses is specific for envelope.

Although a discordance between CD4 T cell and Ab responses to envelope was rather surprising, these findings are consistent with recent studies on chronic lymphocytic choriomeningitis virus (LCMV) infection in mice demonstrating that partial removal or tolerization of virus-specific CD4 T cell responses actually enhanced anti-viral Ab responses [32,33]. Likewise, blocking of CD27-mediated signaling on CD4 T cells to prevent the secretion of IFN-γ and TNF-α, elicited neutralizing Ab responses against LCMV and reduced virus burden [34]. One explanation proposed to explain this phenomenon is that high levels of inflammatory cytokines produced by T cells, including IFN-γ and TNF-α, during a chronic virus infection causes lymphoid tissue damage and retards the capacity of B cells to produce high-affinity Abs effective against the virus. Nevertheless, the loss of CD4 T cell responses during a chronic LCMV infection was also shown to result in the failure to mount Ab responses to escape variants, indicating that a proper balance between CD4 T cell and Ab responses is crucial for effective anti-viral immunity. Another possibility is that rather than the quantity, it is the quality of the CD4 T cell responses which determines the optimal anti-viral Ab responses, although the specific quality or functions required remain unclear.

## Conclusion

In summary, we have demonstrated that the binding of Abs to the CD4bs have distinct effects on CD4 T cell and Ab responses to HIV-1 envelope antigen *in vivo*. These modulating activities correspond with the capacity of the particular Abs to alter structural conformation and proteolytic processing of the envelope antigen. A better understanding about the interaction between Ab reactivity and CD4 T cell recognition of HIV-1 antigens is needed for developing more effective vaccines against the virus.

## Methods

### Immunizations

BALB/c or B10.A mice (female, > 6 weeks old from Jackson Lab) were immunized as previously described [14]. In brief, mice were inoculated i.p. with envelope antigens, alone or with the designated mAb. MPL+TDM (Ribi) or incomplete Freund's adjuvant (IFA) (Sigma-Aldrich, St Louis, MO) were used as adjuvants. When gp120 and mAb were used as immunogens, the immune complexes were prepared by incubating gp120 with the mAb for 2 hrs at 37°C, and then mixed with Ribi adjuvant. Animals were immunized 4 or 5 times at the designated intervals followed by collection of blood and spleens. The animal studies were carried out according to a protocol approved by the VA and NYU IACUC.

### Lymphoproliferation assays

T cell proliferation was assessed using a standard ^3^H-thymidine incorporation assay. To evaluate T cell responses in immunized mice, freshly isolated splenocytes were resuspended in complete RPMI medium supplemented with 2.5% IgG-depleted FBS or with 10% FBS. The cells were then incubated at a concentration of 3 × 10^5^/well either with antigen or with medium alone for 5 days. The cells were pulsed with ^3^H-thymidine for 16–22 hr prior to harvest. Each experimental condition was tested in six replicates.

The gp120-specific mouse CD4 T cell clone T1 was tested for proliferative response as described previously [35]. Irradiated mouse splenocytes used as APCs were treated with gp120 or gp120 complexed with anti-gp120 mAbs, and incubated with the T cell clone. T cell responses to APCs alone in the absence of any antigen were determined in each assay as background proliferation. Each experimental condition was tested in triplicate and all experiments were performed at least twice.

### ELISA for detection of serum Abs

Ig subtypes of gp120-specific Abs in the sera were detected by ELISA as previously described [14]. Briefly, ELISA plates (Immunoblot 2HB Thermo, Milford, MA) were coated with 1 μg/ml sheep polyclonal anti-C5 Ab (Cliniqa, Fallbrook, CA) to capture gp120_LAI _(1 μg/ml). Sera were then reacted for 2 hrs at 37°C. Rabbit Abs against mouse IgG or IgM (Zymed, San Francisco, CA) were added, and alkaline phosphatase-conjugated goat anti-rabbit IgG (Sigma-Aldrich, Saint Louis, MO) was used as a secondary Ab and p-nitrophenyl phosphate (Sigma-Aldrich, Saint Louis, MO) was used as the substrate.

ELISA used to detect anti-CD4bs serum Ab was described previously [9]. Briefly, gp120_IIIB _was captured onto the plate by sheep polyclonal anti-C5 Ab, washed, and reacted with sera. Soluble human CD4 (Progenics) was then added, and bound CD4 was detected using a mouse anti-human CD4 MAb OKT4 and goat anti-mouse IgG Abs conjugated to alkaline phosphatase. Each sample was tested in duplicate wells at least twice.

## Competing interests

The authors declare that they have no competing interests.

## Authors' contributions

MV carried out the immunization experiments with immune complexes and analyzed the induced immune responses, and contributed to writing the manuscript, MT carried out the immunization experiments with envelope variants and performed *in vitro *proliferation assays, PC carried out the immunization experiments with envelope variants and performed ELISA, CEH conceived and coordinated the study as well as drafted the manuscript. All authors read and approved the final manuscript.
